# Synthesis and characterization of a highly stable mucoadhesive gel for oral drug delivery

**DOI:** 10.3389/fdmed.2025.1696639

**Published:** 2025-11-03

**Authors:** Fatima Shahid, Hashmat Gul, Muhammad Amber Fareed, Faheem Ullah, Humayoon Shafique Satti, Muhammad Kaleem

**Affiliations:** ^1^ Department of Dental Materials, Army Medical College, National University of Medical Sciences, Rawalpindi, Pakistan; ^2^ Clinical Sciences Department, College of Dentistry, Ajman University, Ajman, United Arab Emirates; ^3^ Centre of Medical and Bio-allied Health Sciences Research, Ajman University, Ajman, United Arab Emirates; ^4^ Department of Biological Sciences, National University of Medical Sciences (NUMS), Rawalpindi, Pakistan; ^5^ Bioresource Technology Division, School of Industrial Technology, Universiti Sains Malaysia, Gelugor, Malaysia

**Keywords:** mucoadhesive hydrogel, oral ulcers, mesenchymal stem cell derived exosomes, drug delivery, antimicrobial activity, cytotoxicity, biocompatibility

## Abstract

**Objectives:**

This study aimed to synthesize and characterize a novel mucoadhesive hydrogel for oral drug delivery, predominantly for the treatment of oral ulcers. Exosomes derived from mesenchymal stem cells were incorporated into the hydrogel with the intention of enhancing the therapeutic effects and minimizing risks such as drug resistance and secondary infections.

**Methods:**

A basic hydrogel was synthesized via a combination of hyaluronic acid and carbomer. In addition, various antimicrobial agents were added to produce hydrogel variants. Exosomes were incorporated into the hydrogel variants to create a system for drug delivery. Scanning electron microscopy (SEM), Fourier transform infrared spectroscopy (FTIR), a rheological analysis, mucoadhesive testing, and a swelling and degradation analysis at different pH levels were carried out to characterize the hydrogel. *In vitro* testing of the basic composition and the hydrogel variants was then carried out, which included a determination of their antimicrobial properties against *Escherichia coli, Streptococcus aureus,* and *Candida albicans*, a cytocompatibility analysis via a WST-8 fibroblast quantification assay, and an analysis of exosome release kinetics via UV-Vis spectrophotometry.

**Results:**

The SEM analysis revealed a porous and interconnected structure within the hydrogel matrix, which is essential for loading drugs. The FTIR spectrum's characteristic peaks confirmed the presence of the constituent polymers. The hydrogel exhibited suitable viscoelastic properties, strong mucoadhesion on bovine mucosa and satisfactory swelling and degradation properties at various pH levels. Effective antimicrobial activity against *E. coli*, *S. aureus,* and *C. albicans* was observed in the study groups in addition to cytocompatibility and an increase in exosome release from the hydrogel with time.

**Conclusion:**

This study determined that the exosome-loaded mucoadhesive hydrogel is a promising alternative to traditional oral ulcer treatments. The synthesized hydrogel’s viscoelastic and mucoadhesive behavior, along with its antimicrobial activity and biocompatible nature, suggest that it addresses the challenges in conventional oral ulcer treatment.

## Introduction

Exosomes are membranous vesicles that are produced within a cell and then excreted into the extracellular space. Exosomes are being widely used for numerous applications in the field of biomedicine to regulate wound healing through the activation of several pathways. Exosomes have shown the capability to pass the blood–brain barrier; thus, they have the potential to treat brain injuries, including stroke. Likewise, they have shown potential to be used in the treatment of cardiac diseases, hepatic diseases, and even bone regeneration (1). Almost all cells, including epithelial cells, cancer cells, and mesenchymal stem cells (MSCs), can produce exosomes, enabling them to carry out numerous cellular functions, such as communication between cells and responses to various traumas and infections. Exosomes carry critical information regarding the functions of cells as they consist of various proteins, nucleic acids, and lipids (1).

The majority of the research regarding the use of exosomes revolves around their ability to be loaded with drugs, their ability to retain the loaded drug, and the possibility of drug delivery to the target site. Drug delivery systems often exhibit poor delivery to the target site along with poor biocompatibility. However, drug delivery systems based on exosomes are a promising approach to resolve these issues (2). Hydrogels are being used as drug delivery vehicles primarily because of their biocompatible nature. They can be used to protect the drug from the surrounding environment and contribute to a controlled release (3). When made from degradable polymers, the drug is released slowly in the body after degradation of the hydrogel (4). Some strategies for drug delivery include loading hydrogels with exosomes. The cellular compatibility of hydrogels makes them a suitable medium to deliver these extracellular vesicles to the target site (5).

Exosome-based therapy has great application potential in different medical fields, ranging from regenerative medicine to oncology in the craniofacial and dental fields. However, there is limited research on exosomes as a potential treatment for oral ulcers. Oral ulcers usually appear as small, circular lesions with yellow or gray borders and can be caused by various factors (6). Oral ulcers can either be acute or chronic. Acute ulcers are characterized by their short duration, and they heal completely, whereas chronic ulcers can progress into severe disease (6). Acute ulcers are usually caused by infections, reactions to medications, or trauma. Chronic ulcers are associated with diseases such as lichen planus, mycosis, and pemphigus vulgaris (7).

Various topical drugs are available for the symptomatic management, i.e., reduction of pain and duration, of oral ulcers and constitute the first-line treatment (8). Anesthetics, such as lidocaine and benzydamine hydrochloride; anti-inflammatory drugs, such as diclofenac with hyaluronic acid; and corticosteroids, such as dexamethasone and triamcinolone acetonide, are used to treat oral ulcers (8). However, in patients with severe chronic ulcers, immunosuppressive treatment is administered, which has adverse effects in the long term, such as drug resistance, imbalanced oral flora, and being prone to secondary fungal infections (6). Although the current guidelines for managing oral ulcers are effective, treating these lesions is challenging due to the oral cavity's moist environment and dynamic conditions, i.e., food intake, actions of speech, chewing, and swallowing, making it more difficult for topical medications to be retained in the mouth (9, 10). Oral lesions require at least 12–24 h to heal optimally, but these topical ointments and gels are removed by saliva in less than 1 h (11). Newer strategies involve the use of biocompatible hydrogels as drug delivery vehicles to treat oral ulcers (12). Hydrogels are hydrophilic networks made of polymers. They are three-dimensional structures and possess the ability to absorb a significant amount of water or any type of bodily fluid (13). They have a high resemblance to body tissues because of their increased amount of water, porous structure, and flexible nature (14). The presence of a large amount of water in their structure enables them to be used for various applications, especially in the field of medicine (13). Thus, hydrogels that exhibit mucoadhesion are being explored as a possible option to treat oral ulcers. Mucoadhesion is the ability of particles within the hydrogels to breach the mucosal lining. This phenomenon depends on the interaction of the hydrogel with the surrounding environment, resulting in the swelling and expansion of the hydrogel (15).

Carbomer is a polymer that exhibits mucoadhesion. It has been used to deliver drugs efficiently to the mucosa (16). The mucoadhesion exhibited by carbomer is due to the formation of bonds among the carboxyl (–COOH) group of carbomer and mucin, which is a constituent of mucous. Mucin is secreted by the epithelium and consists of glycoproteins. It has a core comprising protein and a side chain consisting of carbohydrates. This is site-targeted to enhance the retention of drugs (16). Carbomer has gained attention as a mucoadhesive polymer due to its biodegradability, biocompatible nature, and good adhesion with biological surfaces. It is also cost-effective (16). Carbomer has also exhibited sustained release of drugs from all its formulations, which is an important characteristic as it helps with patient compliance (16). It is stable up to 260°C (17). Li et al. synthesized a gel containing carbomer, which exhibited good mucoadhesion and swelling properties (18).

Hyaluronic acid has consecutive units of D-glucuronic and N-acetyl-D-glucosamine in its structure. It has a net negative charge because of the carboxyl groups in its structure. It is hydrophilic in nature and forms a highly viscous structure at high molecular weight (19). Its viscoelastic and biological properties are similar to mucin, which allows for a longer retention time (20). Hyaluronic acid exhibits excellent biocompatible properties and degrades inside the body. Due to these properties, it is highly sought after in the field of biomedicine as it can be used for various biological conditions and pathologies. Its biocompatible nature allows it to be used in a number of commercial products, especially cosmetics. It is stable up to 120°C (21). It has also been utilized to synthesize hydrogels for the delivery of drugs and has been an integral tool in tissue engineering (22). Choi et al. synthesized a hydrogel patch using hyaluronic acid that showed excellent osteoconductive potential (23).

Exosome-based therapy and potential biomarkers for various diseases in the oral cavity are widely used in the fields of oncology and regenerative medicine. However, there is limited research on hydrogels loaded with exosomes as a potential treatment option for oral ulcers. Furthermore, there are few studies on mucoadhesive hydrogels loaded with exosomes to treat oral ulcers. Since the novel hydrogel in this study will not be loaded with any antibiotics, this solves the problem of drug resistance. Therefore, this study synthesized a non-toxic, mucoadhesive hydrogel loaded with MSC-derived exosomes for targeted drug delivery in a controlled manner.

## Materials and methods

Hyaluronic acid, carbomer (C_3_H_4_O_2_), carbohydrazide, beta-glycerophosphate were purchased from Shanghai Macklin Biochemical Co., Ltd. (Shanghai, China), and 98% absolute ethanol, ethylenediaminetetraacetic acid (EDTA), and tannic acid were obtained from Sigma Aldrich (St. Louis, MO, USA).

### Preparation of the hyaluronic acid gel

First, 1.25 g of hyaluronic acid was gradually added to 100 mL of deionized water in a solution bottle. The solution was then covered with a lid and placed on a hot plate stirrer and stirred at 650 rpm for 3 h at room temperature (25°C) to form a hyaluronic acid gel.

### Preparation of the carbomer gel

First, 0.4 g of carbomer was added to 50 mL of deionized water and the mixture was allowed to settle in the bottle, followed by stirring at 1,000 rpm for 2 h at room temperature (25°C) on a hot plate stirrer. To neutralize the carbomer gel, two pellets of NaOH, each weighing 0.164 g, were then added, followed by stirring of the gel for another 30 min with a lid on the bottle.

### Preparation of mucoadhesive gel

**Figure 1:**
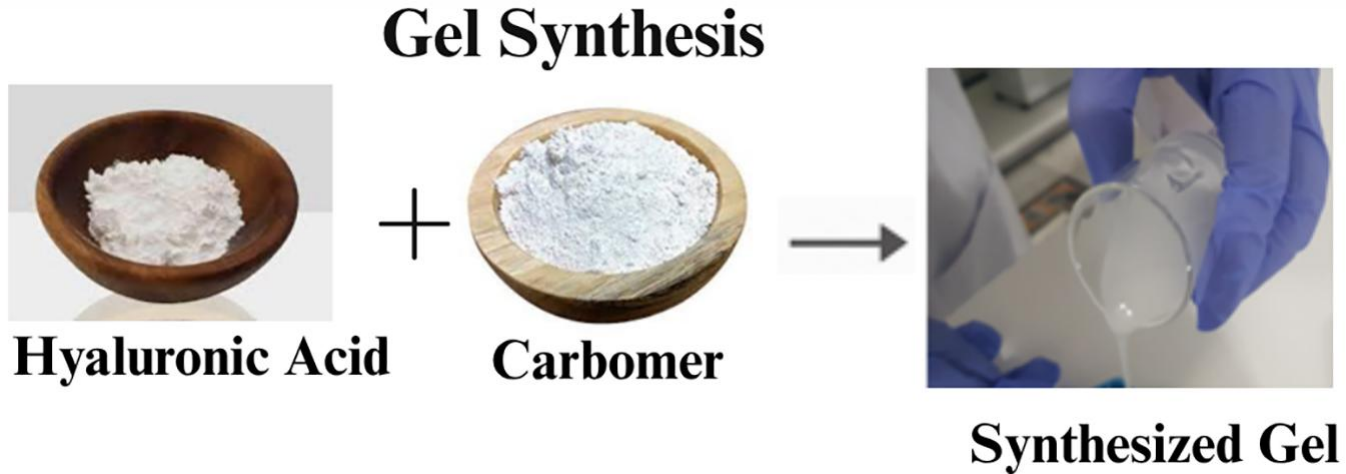
Schematic illustration of the synthesis of the hydrogel.

First, 4 mL of hyaluronic acid gel in a glass beaker was stirred at 300 rpm at room temperature (25°C) on a hot plate stirrer for 1 min, followed by the addition of 1 mL of 20% ethanol and 100 µL of pumpkin oil. The mixture was then stirred for another 5 min, followed by the addition of 2 g of carbomer gel to the mixture. The mixture was then stirred for another 3 min at 850 rpm to yield a homogenous mucoadhesive gel (24). The formulated gel is shown in Figure 1.

### Preparation and grouping of the gel variants

The antimicrobial agents used to create the hydrogel variants included EDTA, tannic acid, β-glycerophosphate, and carbohydrazide. To prepare the hydrogel variants, first, the required antimicrobial agent solutions were prepared by dissolving 0.5 g of the antimicrobial agent powder in 5 mL of deionized water in a beaker covered with aluminum foil, which was then stirred at 650 rpm for 30 min at room temperature (25°C) on a hot plate stirrer. Then, 150 µL of the prepared antimicrobial agent solution was added to 2 mL of the basic composition gel in a beaker and stirred again at 400 rpm for another 2 min on a hot plate stirrer at 25°C to prepare the variant hydrogel group. The details of the prepared hydrogel groups used in this study are given in Table 1.

**Table 1:** Hydrogel variant groups.

S. No.	Modification	Group
1	Basic gel	CG (positive control)
2	Basic gel + EDTA	CG1
3	Basic gel + tannic acid	CG2
4	Basic gel + ß glycerophosphate	CG3
5	Basic gel + carbohydrazide	CG4
6	Carbomer gel	C (negative control)

EDTA, ethylenediaminetetraacetic acid.

### Loading the hydrogel with exosomes for *in vitro* release analysis

Exosomes derived from MSCs were isolated using ultracentrifugation. A fresh exosome solution was then added to the freshly prepared hydrogel or its variant after gelation was visually observed. This was followed by stirring on a hot plate stirrer at 800 rpm for 3 min at 25°C.

### Dehydration of the prepared basic hydrogel for SEM and FTIR

The hydrogel was placed in a hot air oven at 70°C and normal atmospheric pressure for 3 h and then kept at 40°C and normal atmospheric pressure for 24 h to yield dehydrated samples of the basic hydrogel. The dried hydrogel samples were then ground into a powder form with a mortar and pestle (24).

### Scanning electron microscopy

A sputter coater (Q150/S by Quorum Technologies, UK) was used to sputter coat the synthesized hydrogel powder sample with gold particles. A field emission scanning electron microscope (FESEM; MIRA, Tescan, Czech Republic) was used for analysis of the surface features and microstructures of the gold sputter-coated hydrogel powder sample at different magnifications, while being fixed to SEM stubs (25).

### Attenuated total reflection–Fourier transform infrared spectroscopy

An attenuated total reflection–Fourier transform infrared (ATR-FTIR) spectrometer (Thermofisher Nicolet Summit Pro, USA) equipped with OMNIC Paradigm evaluation software was used to analyze the different functional groups present in the synthesized mucoadhesive hydrogel powder samples to determine the change in chemical structure and molecular interactions. The background was captured to remove any noise peaks. The transmittance spectra were obtained in the range of 4,000 to 400 cm^−1^ with a resolution of 4 cm^−1^.

### Rheological characterization

The rheological analysis of the hydrogel samples was carried out using an Anton Paar Physica MCR 301 rheometer (Austria) to describe their internal cross-linked microstructures. The samples were analyzed in gel form. The conditions of the experiment were constant throughout the measurement procedure. The findings were obtained by connecting the rheometer to the PP25 measurement system (parallel-plate measuring system with a 1 mm gap), which has a 25 mm diameter. Every measurement was performed by removing any surplus material and loading a new sample between the parallel plates with a gap value of 1 mm. An oscillatory frequency sweep was conducted using 16 measuring stations at different temperatures (25°C, 30°C, 35°C, and 40°C) throughout the frequency range of 0.01–100 rad s^−1^. The shear stress and viscosity as a function of shear rate were measured using 25 measuring stations and the range of shear rate parameters was 0.1 to 100 s^−1^.

### Bioadhesion

The adhesion of the mucoadhesive hydrogel was determined using Teng and Ho's falling liquid film perfusion technique (26). The test was customized and buccal mucosa from a goat’s head was utilized. The head was freshly bought and stored in an icebox for easy transport. The mucosa was separated using a periosteal elevator and a surgical scalpel. It was then thoroughly rinsed to remove all the blood. The mucosa was then mounted on a glass slide and the mucoadhesive hydrogel was applied to it. Then, physiological saline was introduced drop by drop on the surface of the mucosa using a Luer connector. The flow of the saline was adjusted to 0.04 mL/min to mimic the flow of saliva. The liquid was collected in a glass beaker and 2 mL of the liquid was collected in an Eppendorf tube after every minute initially and then at different time intervals. The liquid was analyzed using a total of 11 different time intervals, i.e., 1, 2, 3, 4, 5, 7, 15, 30, 60, 120, and 180 min. The presence of the gel in each sample was analyzed via UV-Vis spectroscopy. The absorbance was observed at ∼211 nm, which was the absorbance obtained after analyzing a sample of pure gel using UV-Vis. This value corresponds to the presence of hyaluronic acid in the hydrogel (27).

### Swelling/degradation behavior

The fluid uptake capacity of the prepared hydrogel was measured by the swelling measurement. The weight of the dried hydrogel sample/film, 1.5 cm × 1.6 cm in size, was determined using a digital weighing balance. The whole film was then submerged in three buffer solutions of pH 1, 7, and 12, respectively, at room temperature to analyze the swelling/degradation at different pH levels. The hydrogel samples were then removed at different time intervals; the excess water was removed using filter paper, and the hydrogel samples were weighed again.

### Antibacterial testing

To determine the antibacterial properties of the hydrogel and its variants, a turbidity test was performed, following ISO 15522:1999. A nutrient broth was prepared before each antibacterial analysis (28). To perform the antibacterial test, the nutrient broth was mixed with deionized water as per the ratio specified by the manufacturer, i.e., 13:1000. The prepared broth was then sterilized in an autoclave for 15 min at 121°C. Diluted *Streptococcus aureus* and *Escherichia coli* were added to the broth using a micropipette. This process was standardized by measuring the optical density of the microdiluted broth using a UV/VIS spectrophotometer (Biobase, Germany). The wavelength of the spectrophotometer was adjusted to a standard 600 nm (OD_600_). The prepared microdiluted nutrient broth's absorbance was set at a standardized 0.015% at OD_600_ and 100% transmittance. The prepared hydrogel and its variants were placed on the microplate. The microdiluted broth was then added to each of the wells, followed by incubation at 35°C for 24 h. Upon removal from the incubator, the microdiluted broth on the microplate was visually inspected for turbidity. To quantify the results obtained, the optical density of each sample was noted. Blank broth with no bacteria, 0.015% absorbance, and 100% transparency at OD_600_ was set as the standard against which the absorbance of all the samples was measured.

### Antifungal testing

As per ISO/TS 16782:2016, the Kirby–Bauer disc diffusion susceptibility protocol was followed to examine the antifungal effect of prepared hydrogels and the control group, i.e., the carbomer gel. The test was performed in triplicate independent replicates (R1, R2, and R3) on separate plates against *Candida albicans* (*n* = 3). Nutrient agar (Oxoid, UK) was prepared according to the cited procedure and autoclaved at 121°C for 25 min (29). The prepared agar was then poured into sterile Petri dishes and hardened at room temperature (25°C). Then, 15 µL of an overnight-cultured *Candid*a species was spread using an inoculation loop on a separate agar plate. The carbomer gel and prepared hydrogel samples were added in such a way that the coated side faced the fungal lawn.

### Cytotoxicity analysis

A cytotoxicity analysis was performed following the ISO 10993-5 guidelines. To assess the cytocompatibility of the hydrogels via a WST-8 assay, first, the culture medium was prepared, which contained 9.9 mL of Dulbecco's modified Eagle's medium (DMEM; Gibco, USA), 10 vol% fetal bovine serum (FBS; Gibco, USA), and 1 vol% penicillin/streptomycin (Pen/Strep; Gibco, USA). Fibroblast cells were then inoculated and allowed to mature in the culture medium at 37°C and 5% CO_2_ for 48 h in a CO_2_ incubator (Nuaire, UK). Then, 5 × 10^4^ fibroblast cells were counted using a hemocytometer after trypsinization using trypan blue (Sigma Aldrich, USA).

The hydrogel groups were cultured with 5 × 10^4^ fibroblast cells, which were allowed to grow on both control and prepared hydrogels for 48 h in a CO_2_ incubator at 37°C and 5% CO_2_. After removing the culture medium and rigorous washing with phosphate-buffered saline (PBS), 1% WST-8 (Elabscience, USA) in PBS (as instructed by the manufacturer) was added to each well. The absorbance of all the samples was determined in triplicate using a microplate reader (Accuris MR9600 SmartReader 96, USA) at 450 nm. The control sample was given the absorbance value of 100% and the remaining samples were evaluated in comparison to the control.

### Exosome release kinetics

#### Basic composition hydrogel (pilot study)

A pilot study was conducted to study the release kinetics of the exosomes from the hydrogel with the basic composition. Pure hydrogel with no exosomes was used as the control, i.e., Group 1. Fresh exosomes were isolated from MSCs via ultracentrifugation. For the exosome-loaded formulations, a freshly prepared hydrogel (6.88 g) was allowed to visually gel and then mixed with exosome solution as follows: Group 2: 1.50 mL exosome solution added; Group 3: 0.75 mL exosome solution added. This was followed by stirring at 800 rpm for 3 min at 25°C. The three prepared groups for this test were then stored in Falcon tubes at 4°C in an icebox until further use.

The evaluation of the release kinetics of the exosomes from the prepared hydrogel groups was conducted using UV-visible spectroscopy by utilizing the dissolution method using PBS with pH 7.4 (30). Then, 1 g of exosome-loaded hydrogel was added to 15 mL of buffer solution in a beaker with constant agitation at 50 rpm using a magnetic stirrer at room temperature. Subsequently, 2 mL of elutes was removed at planned time intervals, i.e., 1, 2, 3, 4, and 5 min, followed by replacement with an equal volume of PBS at each interval. Finally, a UV spectrophotometer was used to measure the amount of exosomes released at the time intervals in the collected elute samples (31). The absorbance was measured at 291 nm after a solution containing exosomes showed a peak at 291 nm. This can be attributed to the various proteins present in the structure of exosomes, which show absorbance in the range of 280–300 nm (32). To ensure reliability and reproducibility, the test was conducted three times.

#### Hydrogel variants

After the results of the pilot study were obtained, the hydrogel group with 1.5 mL of the exosome solution was selected. First, 1.5 mL of exosome solution was then added to each modified hydrogel (Table 1) after gelation was confirmed visually, followed by stirring at 800 rpm for 3 min at 25°C. The evaluation of the exosomes’ release kinetics in the experimental groups was then conducted in triplicate using UV-visible spectroscopy by employing the dissolution method, as outlined above (30).

## Results

### Scanning electron microscopy

**Figure 2:**
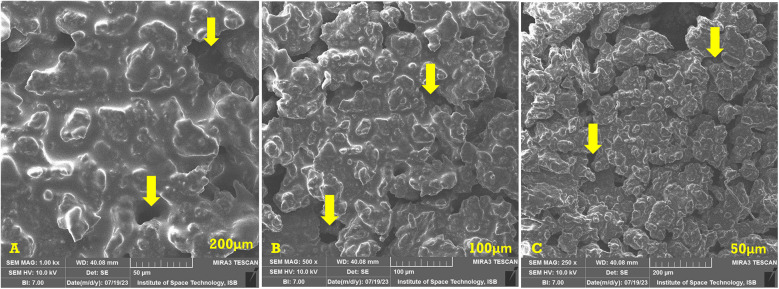
SEM images of the basic hydrogel. The yellow arrows indicate the pores in the hydrogel. **(A)** Magnification of ×1,000, bar = 50 µm. **(B)** Magnification of ×500, bar = 100 µm. **(C)** Magnification of ×250, bar = 200 µm.

The SEM analysis of the basic hydrogel revealed a porous structure with a gel-like matrix, as shown in Figure 2. The surface appeared irregular and rough, and provided a good medium for drug interactions. The surface of the hydrogel appeared aggregated due to the presence of carbomer, whereas hyaluronic acid provided a fibrous structure.

### EDS analysis

**Figure 3:**
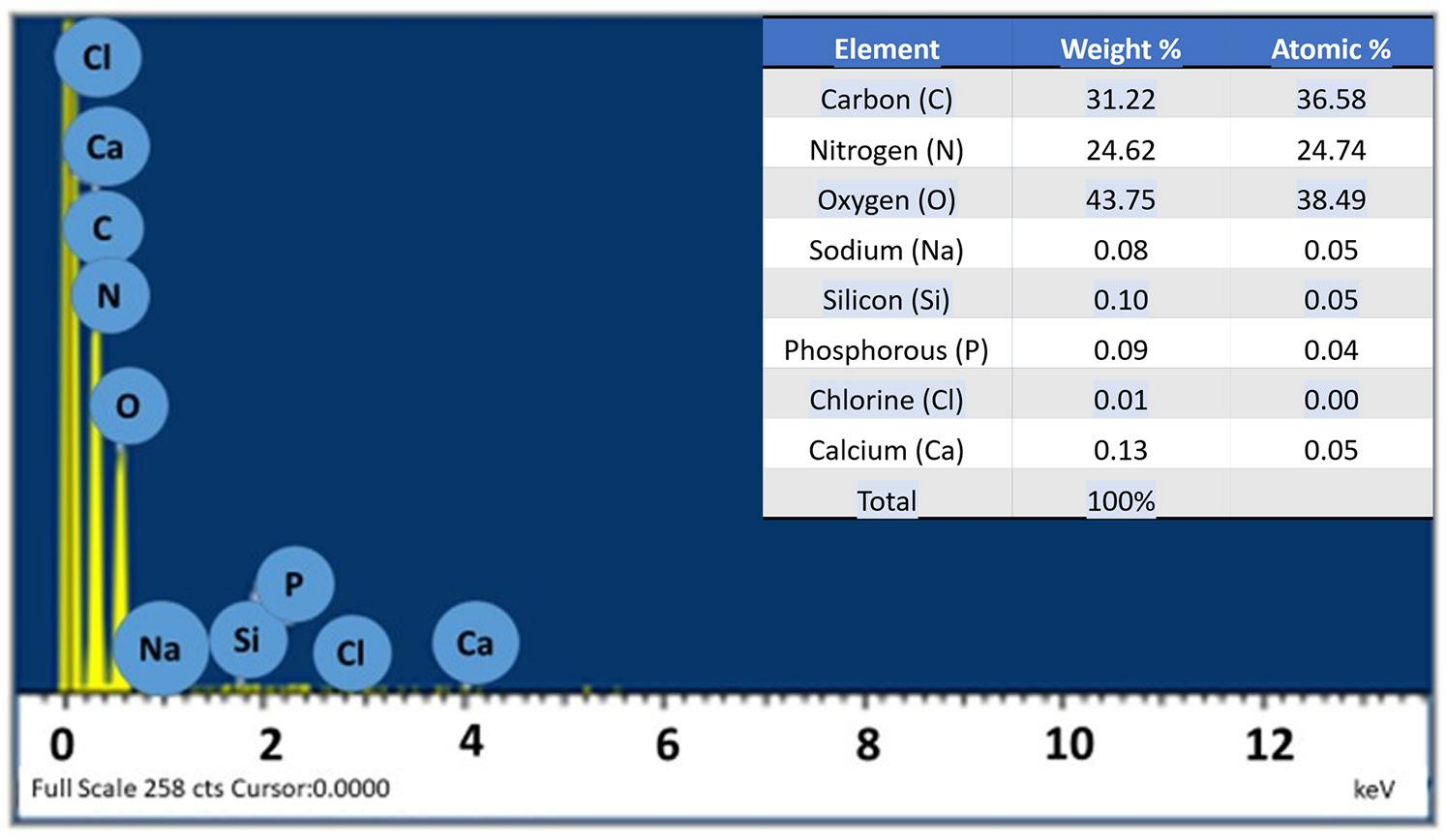
EDS analysis of the hydrogel.

Figure 2 shows the energy dispersive x-ray spectroscopy (EDS) (LEO 435VP, Carl Zeiss™ AG (Jena, Germany) spectrum of the prepared hydrogel which confirms the presence of carbon, nitrogen, oxygen, sodium, silicon, phosphorus, chlorine, and calcium in it. The percentages of the various elements present in the hydrogel are also shown in Figure 3 as well.

### Fourier transform infrared spectroscope

**Figure 4:**
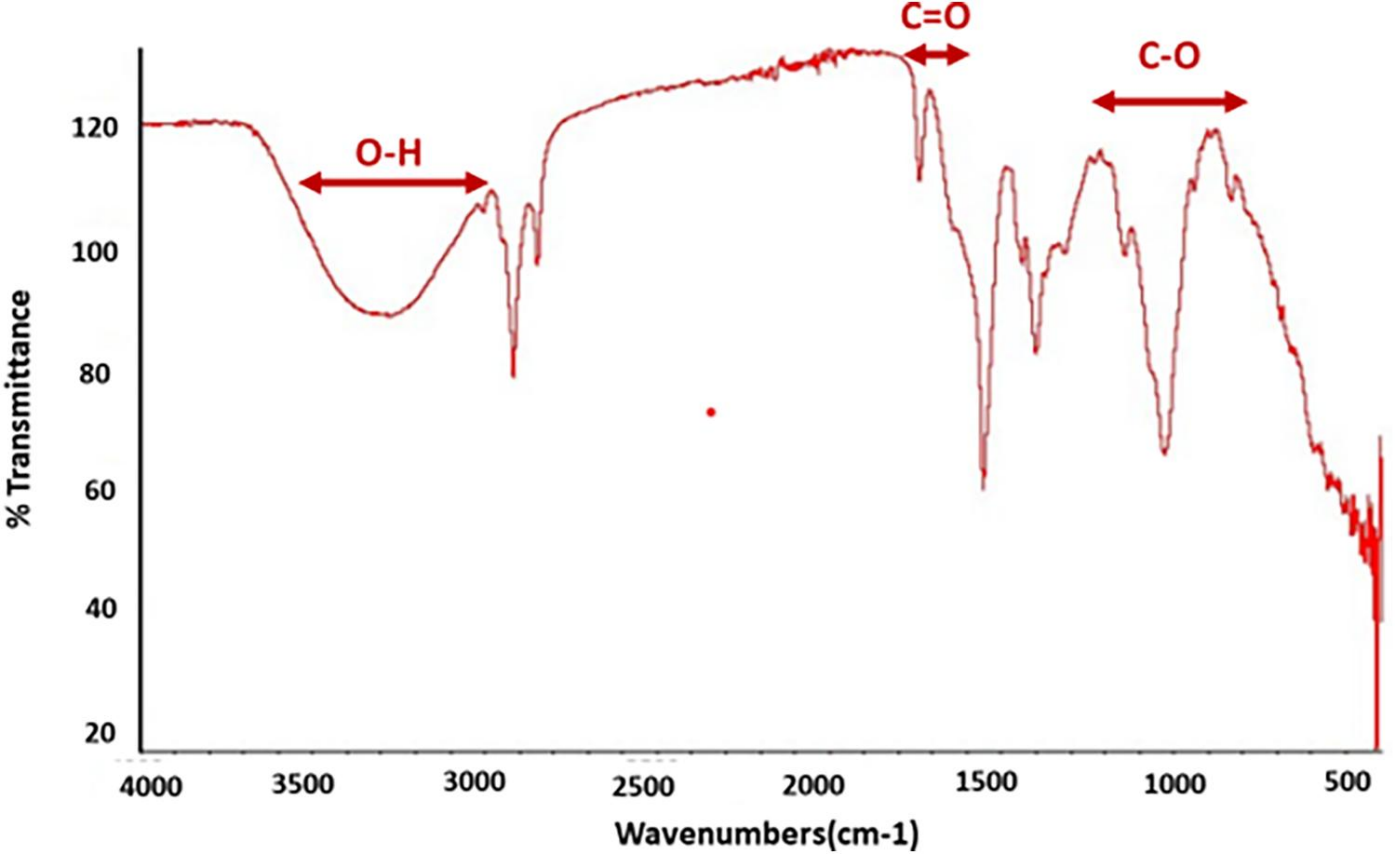
FTIR spectra of the prepared basic hydrogel.

FTIR spectra of the prepared basic hydrogel, given in Figure 4, shows a broad band in the region of 3,200–3,600 cm^−1^, which corresponds to O–H stretching vibrations from both the hyaluronic acid and the water in the gel. A sharp peak around 1,725–1,750 cm^−1^, indicates the presence of the carbonyl group (C=O) in carbomer. A band can be seen in the region of 1,000–1,300 cm^−1^, which is associated with C–O stretching vibrations in both carbomer and hyaluronic acid.

### Rheological characterization

**Figure 5:**
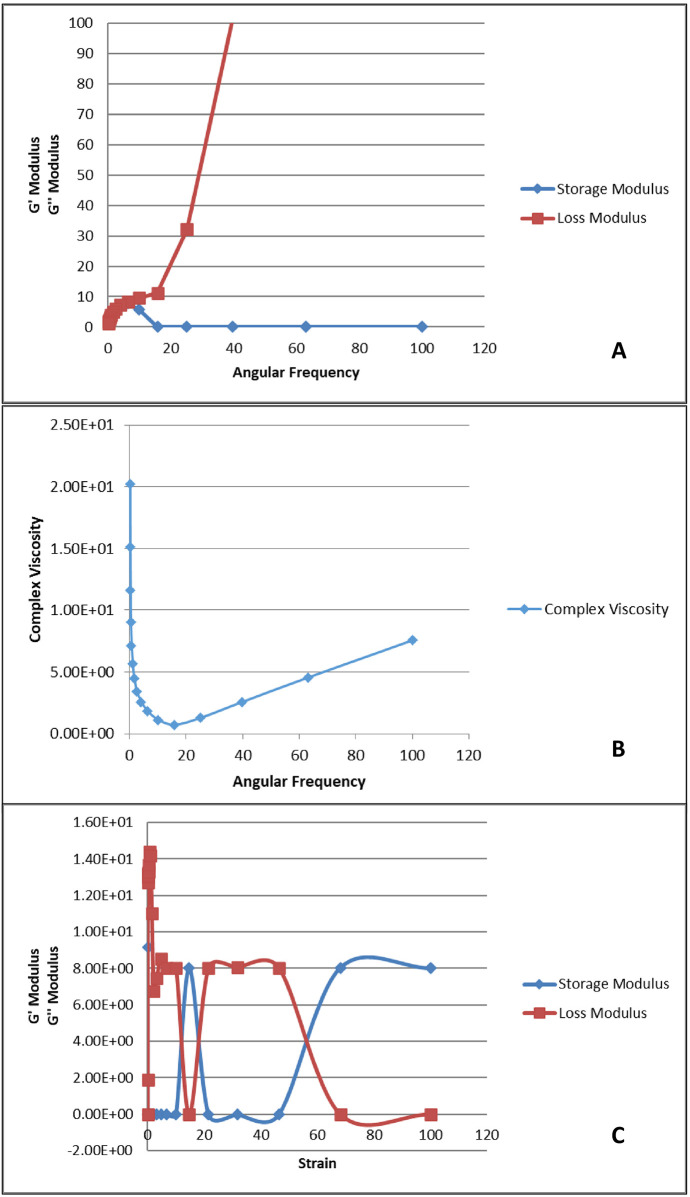
Rheological analysis of the prepared hydrogel. **(A)** Angular frequency vs. storage and loss modulus; **(B)** angular frequency vs. complex viscosity; **(C)** strain vs. storage and loss modulus.

The rheological behavior of the prepared hydrogel is shown in Figures 5A–C. The storage modulus (G') represents the elastic behavior, while the loss modulus (G″) represents the viscous behavior of the hydrogel. In Figure 5A, G″ increases with increase in angular frequency, however, G' is leveled up at zero. This shows that the material is more fluid like and viscous as it is strained, thus, has weaker elasticity and tends to be more fluid/viscous. In Figure 5B, it can be observed that as the angular frequency is increased, the elastic behavior of the sample is overcome which is observed by the relative drop of complex viscosity over the range of 0–20 angular frequency. Once elasticity of sample is no more, the viscous behavior becomes dominant, and the complex viscosity begins to increase. Figure 5C depicts that as strain is increased the elastic behavior of the sample is overcome. This is observed by the relative drop in storage modulus over the range of 0–60 angular frequency. Once elasticity of sample is no more, the viscous behavior becomes dominant and increase in G″ loss modulus dominates.

### Bioadhesion

**Figure 6:**
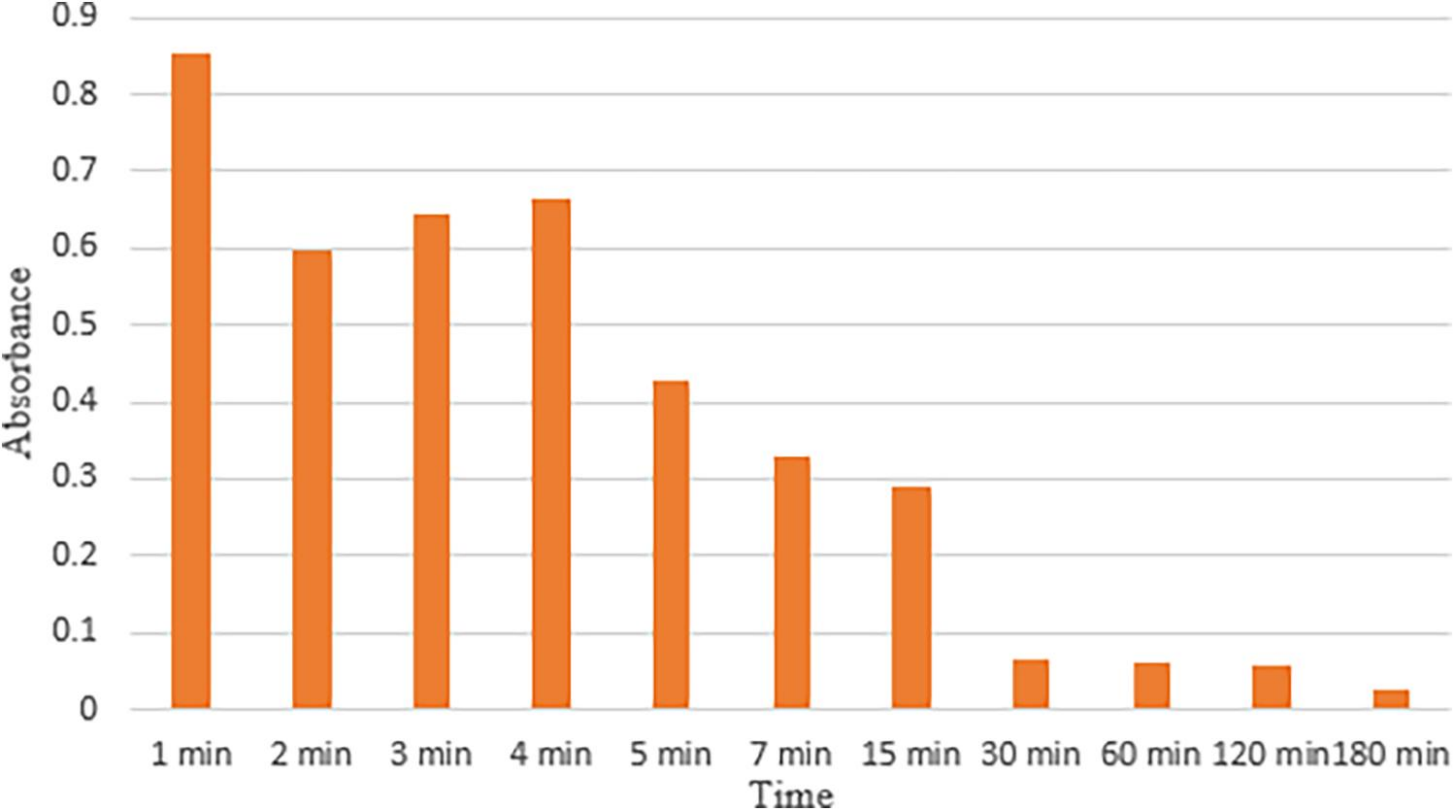
Adhesion of the hydrogel to mucosa.

The hydrogel depicts strong adhesion with mucosa. Figure 6 depicts the values of absorbance at each time interval indicating bioadhesion of basic hydrogel to mucosa. The absorbance decreased over time with the highest absorbance value recorded in the first minute (0.85283 ± 0.935268) and lowest absorbance value at 180 min (0.02617 ± 0.028903).

### Swelling and degradation of basic hydrogel

**Figure 7:**
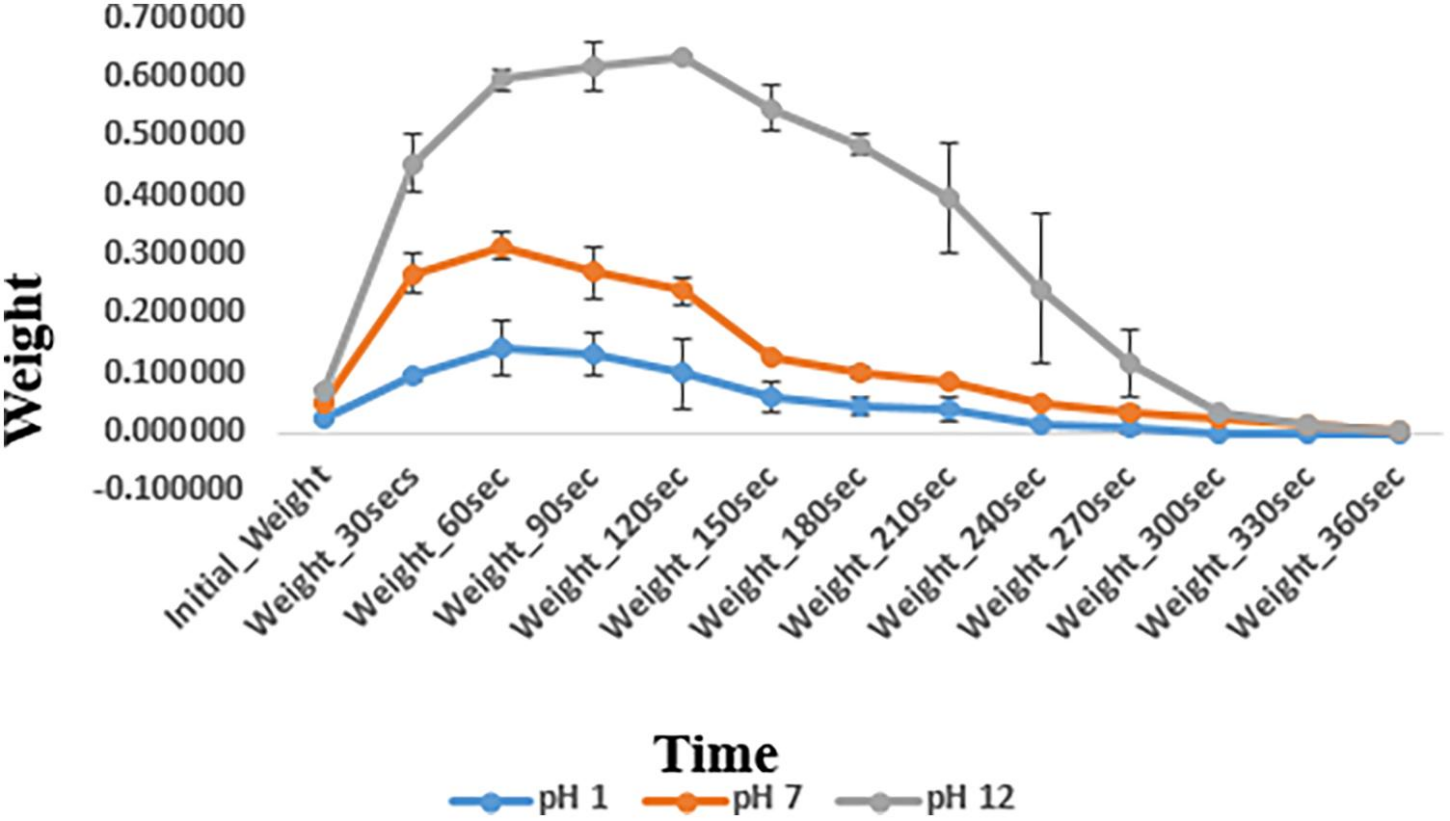
Swelling and degradation analysis at different pH levels.

The swelling and degradation behavior of the basic hydrogel indicated by changes in weight of hydrogel (mean ± standard deviation) at different pH levels is depicted in Figure 7. The swelling/degradation pattern of hydrogel at pH 1 group and pH 7 group is parallel i.e., the hydrogel showed increase in weight up to 60 s indicating swelling/water uptake, followed by a gradual drop in weight, indicating its degradation in 360 s. In pH 12 group, the hydrogel swelled for 120 s and then degraded eventually. Degradation of the hydrogel at different pH also shows a similar pattern. The synthesized hydrogel degraded within 360 s at all three pH groups i.e., pH 1, 7, and 12. A statistically significant difference in swelling and degradation behavior of the hydrogel indicated by changes in weight of hydrogel, was observed within and between different pH groups at planned time intervals. Pairwise comparison showed a statistically significant difference in weight of the hydrogel at planned time intervals between different pH groups (*p*-value <.05) except between pH 1 and pH 7 groups (*p*-value > 0.05).

### Antibacterial activity against *E. coli*

**Figure 8:**
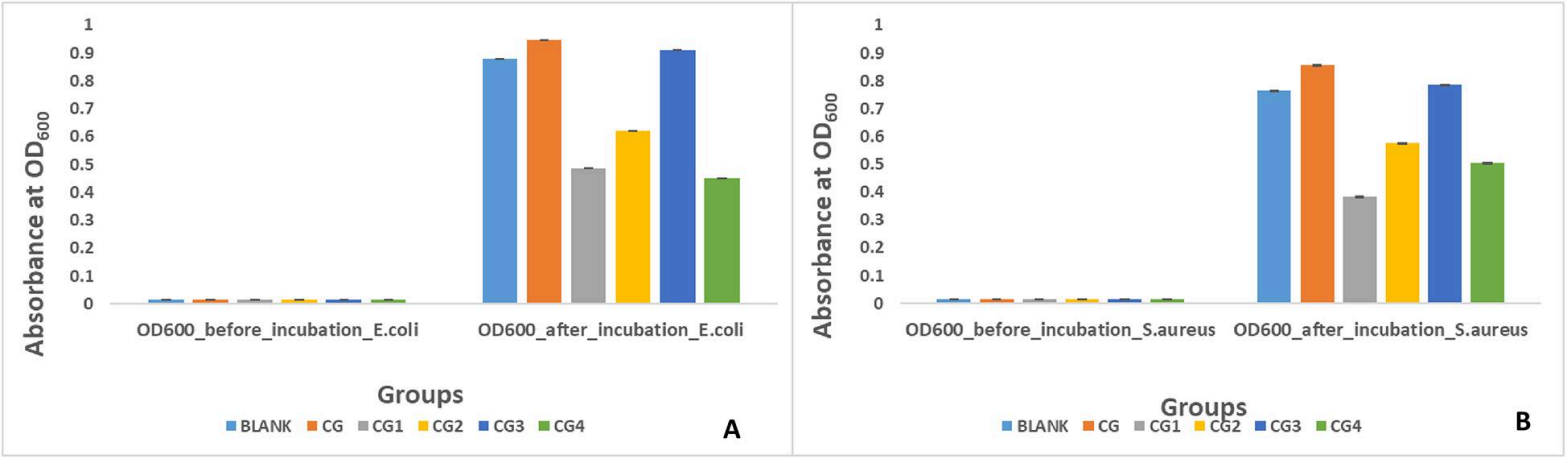
Antibacterial activity of the hydrogel variants against **(A)** *E. coli* and **(B)** *S. aureus*.

Antibacterial activity against *E. coli* was exhibited by three out of the five variants of the prepared hydrogels as shown by turbidity/absorbance at 600 optical density in Figure 8A. Highest antibacterial activity against *E. coli* was exhibited by group CG4 (0.45 ± 0.04), followed by group CG1 (0.48 ± 0.02), and group CG2 (0.62 ± 0.02). A statistically significant difference (*p*-value < 0.05) in antibacterial activity of the hydrogels against *E. coli* was observed within and between all the experimental hydrogel groups. Pairwise comparison showed a statistically significant difference in antimicrobial activity against *E. coli* between all hydrogel groups used in this study except between blank and CG, blank and CG3, CG and CG3, and CG1 and CG4 (*p*-value > 0.05).

Antibacterial activity against *S. aureus* was exhibited by three out of the five variants of the prepared hydrogel tested in this study as shown in Figure 8B. Highest antibacterial activity against *S. aureus* was exhibited by CG1 (0.38 ± 0.01), followed by CG4 (0.50 ± 0.02), and CG2 (0.57 ± 0.01) as shown by turbidity/absorbance at 600 optical density. A statistically significant difference (*p*-value < 0.05) in antibacterial activity of the hydrogels against *S. aureus* was observed within and between all the experimental hydrogel groups. Pairwise comparison of the tested hydrogels showed a statistically significant difference in antibacterial activity between the groups except between groups blank and CG, blank and CG2, blank and CG3, CG and CG3, CG1 and CG2, CG1 and CG4, and CG2 and CG4 (*p*-value > 0.05).

### Antifungal activity

**Figure 9:**
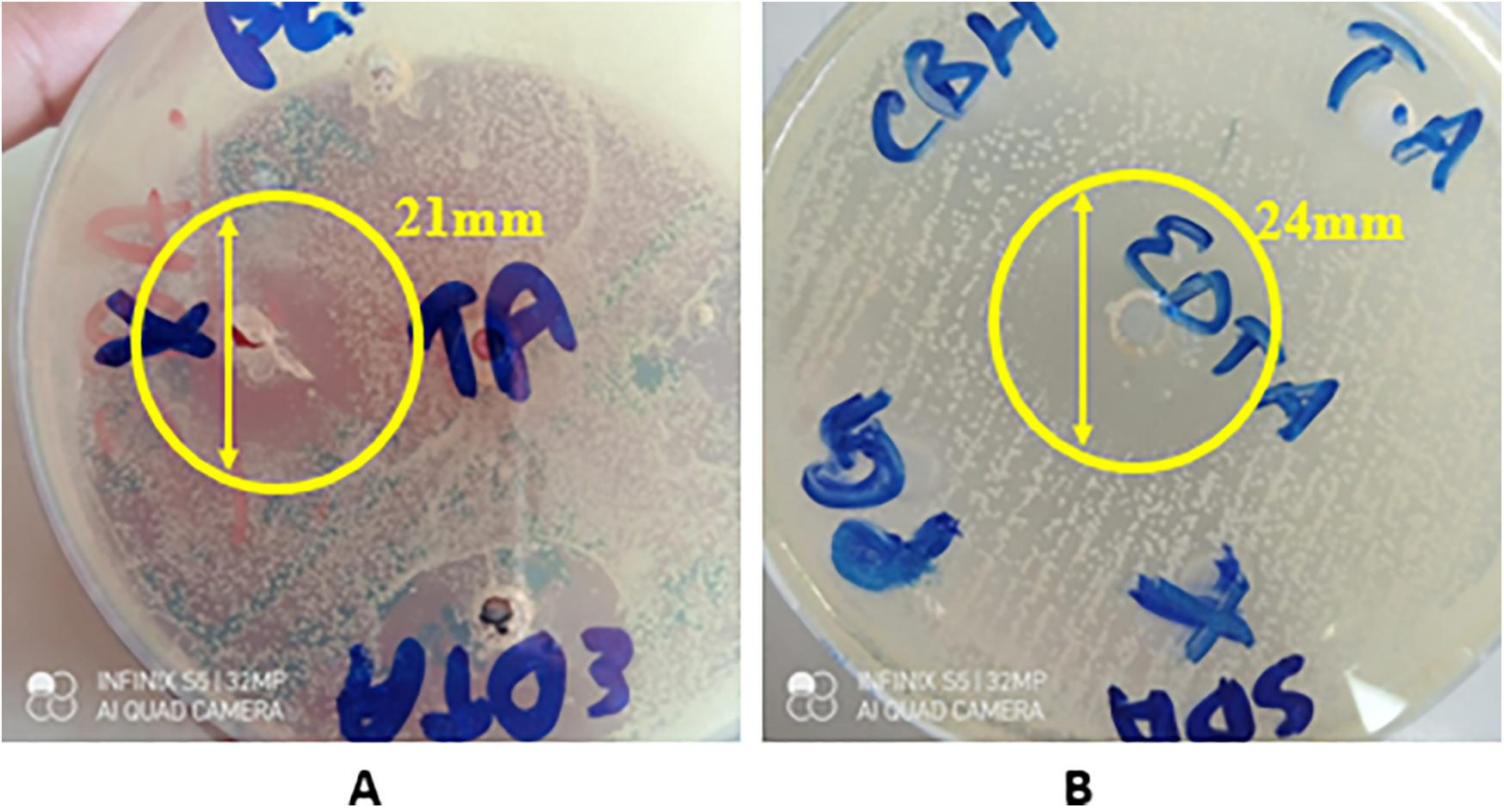
Zones of inhibition against *C. albicans* of **(A)** CG and **(B)** CG1 (*n* = 3).

The disk diffusion test showed that only two out of five variants of the synthesized hydrogel exhibited a strong antifungal effect against *C. albicans* indicated by zones of inhibition (ZOIs), i.e., CG (20.67 ± 2.52) and CG1 (24.00 ± 3.61), as shown in Appendix A and Figure 9. The other three variants failed to produce any zones of inhibition. A statistically significant difference (*p*-value < 0.05) in antifungal activity of the hydrogels, was observed within and between the experimental groups. Pairwise comparison showed a statistically significant difference in antifungal activity of the hydrogels against *Candida albicans* between all study groups except between groups CG and CG1, CG2 and CG3, and CG2 and CG4 (*p*-value > 0.05).

### Cytotoxicity analysis

**Figure 10:**
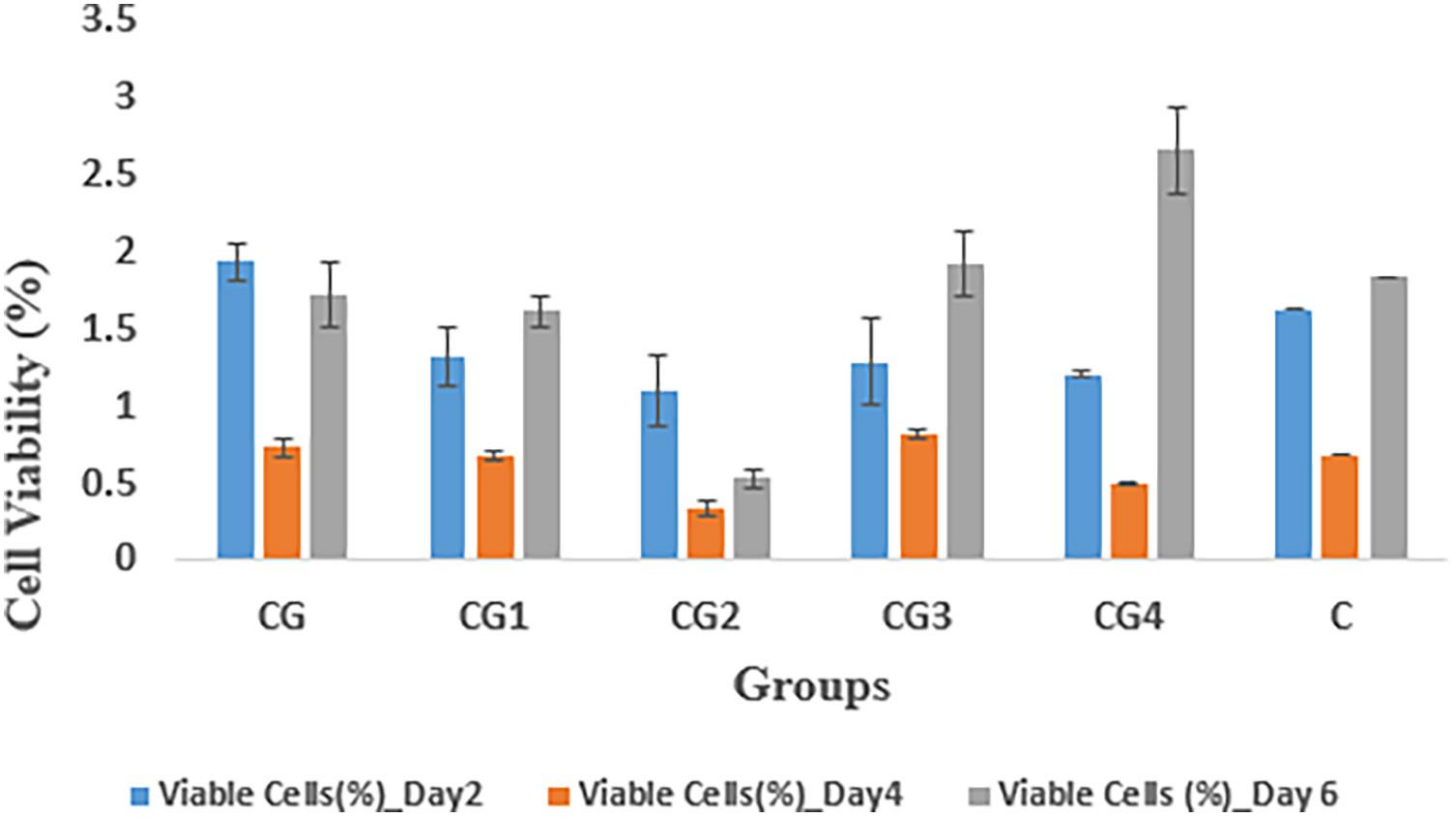
Cytotoxicity analysis of the hydrogel variants.

The cytocompatibility of the hydrogel groups as indicated by percentage of viable fibroblasts against the prepared samples, was evaluated by measuring absorption values at various time intervals i.e., at days 2, 4, and 6, as shown in Figure 10. On 2nd day, group CG exhibited the highest cell viability (1.93 ± 0.12) while CG2 exhibited the lowest cellular viability (1.09 ± 0.23) when compared to control C (1.62 ± 0.00). On 4th day, the highest cell viability was expressed by CG3 (0.81 ± 0.03) while CG2 (0.33 ± 0.05) exhibited the lowest cellular viability when compared to control C (0.68 ± 0.00). On the 6th day CG4 exhibited the highest value of cellular viability (2.65 ± .28), while CG2 exhibited the lowest value of cellular viability (0.53 ± 0.06) when compared to control C (1.83 ± 0.00). In this study, the tested hydrogel groups showed statistically significant (*p*-value < 0.05) difference in cytocompatibility within and between the groups. Pairwise comparison showed a statistically significant difference in cytocompatibility only between hydrogel groups CG and CG2, CG1 and CG2, CG2 and CG3, and CG2 and CG4 (*p*-value < 0.05), however, the rest of the tested hydrogel groups showed no statistically significant difference between the groups (*p*-value > 0.05).

### Exosomes release kinetics

#### Release from basic composition hydrogel

**Figure 11:**
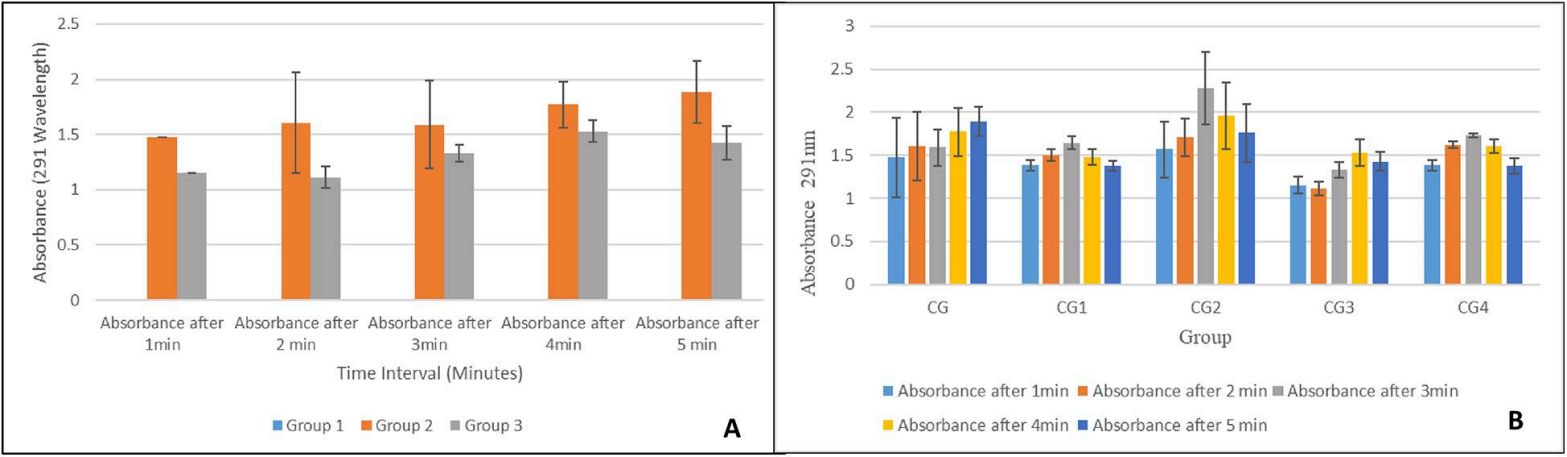
Exosome release from **(A)** the basic composition hydrogel and **(B)** the hydrogel variants.

In the pilot study, increased exosome release was observed considerably by both exosomes loaded hydrogel groups i.e., group 2 and group 3 when compared to control/exosome-free hydrogel i.e., group 1 as shown in Figure 11A. Both exosomes loaded groups showed a similar trend of increasing absorbance values over time. Group 2 with 1.5 mL fresh exosomes exhibited higher absorbance values as compared to the group 3 with 0.75 mL fresh exosomes, thus, showing direct proportionality of exosomes release with exosome concentration in hydrogel. A statistically significant difference (*p*-value < 0.05), in exosome release, was observed within and between the hydrogel groups, however, pairwise comparisons showed a statistically significant difference (*p*-value < 0.05) in exosome release between all groups at every time intervals except between group 2 and group 3 (*p*-value > 0.05).

All modifications/variants of the hydrogel showed a similar trend of exosome release as shown in Figure 11B. The highest exosomes release was observed at the third minute for most of the hydrogel groups and then there was a gradual decline. Group CG, however, depicted a continuous increase in exosome release over time. Group CG2 showed the highest exosome release at each interval. Pairwise comparison showed no statistically significant difference in exosome release between all hydrogel groups except between group CG2 and group CG3 (*p*-value = 0.05).

## Discussion

Oral ulcers are painful lesions in the oral cavity due to disruption of the epithelial lining of the mucosa. These lesions can significantly impact patients’ quality of life as they cause a constant feeling of discomfort (6). Oral ulcers, whether acute, chronic, or recurrent, are caused by numerous factors such as infections, physical trauma, or any systemic conditions (7). The constant discomfort caused by oral ulcers influence many daily activities such as eating, speaking and swallowing which is why effective treatment is imperative to provide relief to the patients (11). Conventional treatment for oral ulcers is focused on managing symptoms and reducing discomfort caused to the patients. Somogel, a widely used topical drug, provides localized relief due to its anesthetic properties. Similarly, corticosteroids, analgesics, and antiseptics are also prescribed to treat oral ulcers. Although these topical drugs are effective, there are certain limitations associated with their treatment such as short retention time due to the dynamic environment of the oral cavity or resistance to drugs. Somogel, though more retentive acts mostly by relieving pain due to anesthetic agents in its composition (6, 11).

To achieve an ideal therapeutic outcome, treatment for oral ulcers should be biocompatible and should be effective for a prolonged period of time. It should also exhibit anti-inflammatory properties. This has directed attention toward exosomes, which are extracellular vesicles found in almost all cells of the body. Exosomes have shown exceptional biocompatibility and minimal adverse effects due to their organic nature. They promote wound healing by activating numerous pathways and modifying immune response without any of the side effects reported with conventional treatments (1). The applications of exosomes in the biomedical field are extensive. They range from regenerative medicine to treating patients of stroke (1). In the case of oral ulcers, they can be used for targeted drug delivery and prolonged relief. However, delivery of exosomes to the target site is a challenge as they are unstable at room temperature. To deliver exosomes to the targeted site efficiently, they can be incorporated in hydrogels, which serve as a stable medium to deliver drugs to the ulcer site (5).

This study mostly focused on characterizing the hydrogel on its morphological features and fundamental properties such as swelling, adhesion, antimicrobial properties, and mechanical stability. These tests were conducted without incorporating exosomes in the hydrogel to avoid compromising their stability during the testing, as most tests are conducted at room temperature and exosomes require a temperature of −80°C to be stable. For shorter duration, they are stable at 4°C (33). This approach to testing ensures that the synthesized hydrogel is appropriate for delivery of drugs while being biocompatible itself. Exosomes due to their organic nature are biocompatible and have shown antimicrobial properties in various studies (34). Thus, this study aimed at synthesizing a mucoadhesive gel to load exosomes efficiently and deliver to the target site topically.

### Characterization of hydrogel with basic composition

#### SEM analysis

The prepared hydrogel exhibits a porous structure with a gel-like matrix. This porous structure is a desirable characteristic as it allows hydrogels to deliver drugs efficiently. The SEM analysis reveals an irregular and rough surface. This allows greater drug interactions by providing a large surface area for attachment of absorption of drugs. The three-dimensional structure of the hydrogel observed on the surface is due to the presence of carbomer. A similar three-dimensional structure due to carbomer was observed in a study conducted by Hui et al. (35). This structure can serve as a reservoir area for drugs within the hydrogel matrix. The fibrous structure seen in the SEM analysis is due to hyaluronic acid. Hyaluronic acid is a naturally occurring polymer that can provide stability and mechanical strength to the hydrogel. Abbasi Aval et al. observed a similar fibrous structure of hyaluronic acid in their study (36). The porous and rough surface of the prepared hydrogel along with the three-dimensional structure provided by carbomer and fibrous structure of hyaluronic acid suggests a hydrogel with promising features for delivery of drugs.

#### Energy dispersive x-ray spectroscopy

The EDS analysis revealed the presence of carbon (C), nitrogen (N), and oxygen (O) in the prepared hydrogel. These elements are also the main constituents of hyaluronic acid and carbomer (37). The table in Figure 3 presents the amount of each element present in the hydrogel, including trace amounts of other elements such as silicon (Si), sodium (Na), and chlorine (Cl). These elements could originate from impurities, for instance, the presence of silicon could be attributed to silicon dioxide (SiO_2_) or other glass impurities. Overall, the analysis confirms that the elements present in the hydrogel were consistent with the elements found in hyaluronic acid and carbomer (37).

#### FTIR analysis

The FTIR spectrum, as shown in Figure 4, was used to identify the functional groups in the prepared basic composition hydrogel. The broad band in the region of 3,200–3,600 cm^−1^, which corresponds to O–H stretching vibrations, indicated the presence of hydrogen bonds in the –OH group, which could be due to the hyaluronic acid and water present in the hydrogel matrix (38). The band in the region of 1,000–1,300 cm^−1^ is due to C–O stretching vibrations, which are also due to hyaluronic acid (39). The presence of a carbonyl group (C=O) is indicated by a sharp peak around 1,725–1,750 cm^−1^. This group was present due to the acrylic acid component of carbomer (40). Thus, the FTIR spectrum confirms that the expected functional groups, namely, –OH, C=O, and C–O, were present in the prepared hydrogel. These groups indicate the presence of hyaluronic acid, carbomer, and water in the hydrogel.

#### Rheological characterization

A rheological test was conducted to understand the viscoelastic nature of the prepared gel. The gel had viscous properties, but, due to a lack of extensive chemical bonding forces within its structure, it demonstrated fewer elastic properties (41). This can be seen in Figure 5A, which shows a comparison of the storage modulus, G′, corresponding to elasticity, and loss modulus, G″, corresponding to viscous behavior, over ranges of applied angular frequency. G″ increases as the angular frequency increases, which indicates that the hydrogel had weak elastic properties and demonstrated a viscous response predominantly. Lower G′ values indicate that the material did not demonstrate a solid-like nature, but rather a more fluid nature with a higher angular frequency. This behavior makes it suitable for applications where the hydrogel needs to adapt to irregular surfaces, such as drug delivery. This result is further supported by the data presented in Figure 5B, which presents a comparison of how straining affects the storage modulus and loss modulus. The figure shows that the gel exhibited the shear thinning phenomenon as the angular frequency increased. This is shown by the decrease in complex viscosity. This is desirable as it indicates that the gel can be used in various biomedical applications and especially in drug delivery systems. Moreover, there was a recovery of storage modulus, possibly due to the re-establishment of elastic bonds or thickening of the viscous solution. The data in Figure 5C also confirms the same behavior, as G′ decreases as the strain amplitude increases. The increase in G″ shows that the hydrogel exhibited viscous behavior. The combined analysis of G′, G″, and complex viscosity shows that the hydrogel's behavior was dependent on a balance between its elastic and viscous components. The stability of the hydrogel between these states after applying various forces suggests that it is suitable for applications in dynamic environments, particularly as a scaffold for tissue engineering or as an injectable for biomedical applications. Lopez and Richtering studied the effect of pH and concentration of crosslinks on the oscillatory rheological profile of carboxymethyl cellulose gels and found that at lower pHs and crosslink concentrations, the gel displays a strain-thinning behavior with low storage modulus (42). Yan et al. observed a similar phenomenon of shear thinning when applying strain to their ß hairpin peptide hydrogels (43).

#### Bioadhesion

The synthesized hydrogel exhibited strong mucoadhesive properties, as shown by the results. The absorbance values give an insight into how long it takes for the hydrogel to completely detach from the mucosal surface. The high absorbance values recorded after the first minute show that the synthesized hydrogel showed strong adhesion to the mucosal surface. This can be attributed to the strong interactions of the components present in the hydrogel with the mucosal surface. Both hyaluronic acid and carbomer exhibit bonding via van der Waals forces or hydrogen bonding with the mucosal surface (44, 45). The absorbance values decrease over time, suggesting a decrease in the mucoadhesion of the hydrogel. The lowest absorbance values were observed at 180 min. Samiraninezhad et al. observed that their hyaluronic acid-synthesized hydrogels showed exceptional performance as topical drug delivery systems and in the sustained release of drugs (46), which is in accordance with this study’s findings. Rédai et al. determined that their carbomer-synthesized hydrogels exhibited superior adhesion and release of drugs when administered rectally (47), which supports this study’s findings. The sustained contact of the synthesized hydrogel with the mucosal surface suggests its effectiveness for drug delivery, especially in conditions where prolonged retention for the delivery of drugs is required, such as in the oral cavity or gastrointestinal tract.

### *In vitro* analysis of the basic gel

#### Swelling and degradation

The major difference in the swelling and degradation analyses was the time for which the swelling was sustained. At acidic pH 1 and neutral pH 7, the hydrogel exhibited swelling for up to 60 s, following which the process of degradation began. In contrast to this, at pH 12, the hydrogel exhibited prolonged swelling, i.e., for 150 s. A pairwise comparison between the gels indicated that there was a statistically significant difference in the swelling and degradation properties of the hydrogel between pH 1 and pH 12, and between pH 7 and pH 12 (*p*-value < 0.05). This difference in swelling and degradation behavior of the hydrogel at different pH values can be attributed to the state of ionization of the functional groups of carbomer present in the hydrogel matrix. At a high pH, these functional groups exhibit greater ionization, which leads to greater electrostatic repulsion and greater swelling. This suggests that repeated applications of the gel are required in conditions where the pH of the oral cavity falls in the alkaline range (48). However, at pH 1 and pH 7, the hydrogel did not exhibit a statistically significant difference in swelling and degradation properties (*p*-value > 0.05). At pH 1 and 7, the net surface charges on the hydrogel were very similar and thus exhibited similar swelling and degradation behaviors (48). Suhail et al. observed a similar behavior with carbomer hydrogels. These gels exhibited greater swelling at a basic pH (40).

### *In vitro* analysis of variant hydrogels

#### Antibacterial activity

Three of the five hydrogel variants exhibited antibacterial properties against *E. coli.* This was evaluated by measuring the absorbance at 600 nm, where lower absorbance indicated greater antibacterial activity. The results revealed that the highest antibacterial activity was exhibited by group CG4 (0.45 ± 0.04), followed by group CG1 (0.48 ± 0.02) and group CG2 (0.62 ± 0.02). This antibacterial activity can be attributed to the specific antimicrobial components in the hydrogel variants. Group CG4, exhibiting the highest antibacterial activity against *E. coli,* contains carbohydrazide, which is an antibacterial agent, and this explains the antibacterial effect of this variant. These results are in accordance with the study by Deng et al., in which sodium alginate and methacrylate gelatin-based hydrogels modified with carbohydrazide exhibited a strong antibacterial effect against *E. coli* (49)*.* Similarly, the CG1 variant contains EDTA, which is a potent antibacterial agent (50). Soleimanpour et al. discovered that EDTA enhanced the antibacterial action against *E. coli* of their alginate-fibrinogen hydrogel for wound healing (51). The presence of tannic acid is responsible for the antibacterial activity of CG2. Tannic acid has shown great potential as an antibacterial and antiviral agent (52). Jing et al. observed that their silk fibroin and tannic acid hybrid hydrogels exhibited good antibacterial activity against *E. coli* (53). The negative results found for other antimicrobial agents can be attributed to contaminants that may have been present in the sample during testing (54).

The antibacterial activity of the five different hydrogel variants against *S. aureus* was similar, as three of the five variants exhibited antibacterial properties. This was again evaluated by measuring the absorbance at 600 nm, where the lower the absorbance, the greater the antibacterial activity. In this test, CG1 (0.38 ± 0.01) exhibited the highest antibacterial activity, followed by CG4 (0.50 ± 0.02) and CG2 (0.57 ± 0.01). Similar to the activity against *E. coli*, this antibacterial activity can be explained by the addition of various antibacterial moieties. EDTA was added to the CG1 variant, which showed excellent antibacterial activity (50). Soleimanpour et al. discovered that EDTA enhanced the antibacterial action against *S. aureus* of their alginate-fibrinogen hydrogel for wound healing (51). CG4 contained carbohydrazide, which exhibits antibacterial activity, and this explains the antibacterial effect of this variant. These results can again be compared with Deng et al., who synthesized hydrogels from sodium alginate and methacrylate gelatin modified with carbohydrazide. These gels exhibited a strong antibacterial effect against *S. aureus* (49)*.* Similarly, the presence of tannic acid was responsible for the antibacterial activity of CG2. Tannic acid has exhibited excellent antibacterial activity (52). Jing et al. observed that their silk fibroin and tannic acid hybrid hydrogels exhibited good antibacterial activity against *S. aureus* as well (53). The lack of antibacterial activity in the other groups could be due to impurities present in the samples, which may have caused reactions that led to negative results (54).

#### Antifungal activity

The results from the disk diffusion test of the hydrogel variants showed that two of the five variants exhibited antifungal activity against *C. albicans.* CG and CG1 showed notable antifungal activity against *C. albicans.* This was determined by the ZOIs created by these variants. CG1 (24.00 ± 3.61) exhibited the highest antifungal activity, followed by CG (20.67 ± 2.52). This antifungal activity can be attributed to the components of the hydrogel and the antimicrobial agents added to its formulation. The antifungal activity of CG and CG1 suggests that these contain an optimal formulation of the hydrogel that enhances its antifungal activity. CG1 contains EDTA, which has demonstrated antifungal activity against *C. albicans*. This is in accordance with results obtained by Dupont et al., as EDTA inhibited the growth of *C. albicans* in their study (55). Similarly, hyaluronic acid is also known to exhibit antifungal activity. Parolin et al. observed that hyaluronic acid showed significant antifungal activity against *C. albicans* (56). Thus, the presence of hyaluronic acid explains the antifungal activity exhibited by both the variants.

#### Cytocompatibility analysis

The cytocompatibility of the hydrogel variants was determined by calculating the percentage of viable fibroblasts after observing the samples over a period of 6 days, as shown in Figure 9. On day 2, the highest cellular viability was demonstrated by CG and CG2, which exhibited the lowest cellular viability when compared to control C. On day 4, CG3 exhibited the highest cellular viability and on day 6, the highest cellular viability was exhibited by CG4. CG2 consistently exhibited the lowest cellular viability throughout the study when compared to control C and this can be attributed to impurities in the samples either during preparation or during testing, leading to altered results (54). A statistical analysis also revealed significant differences between CG2 and the other groups. The cytocompatibility of the hydrogel and its variants can be attributed to the various components in its formulation. Zhang et al. determined that their zwitterionic hyaluronic acid-based hydrogels exhibited excellent biocompatibility, which increased over time (57). Similarly, Hayati et al. concluded that carbomer hydrogels exhibited biocompatibility and improved tissue perfusion and decreased necrotic tissue in burn wounds (58). Salehi et al. used EDTA in their chitosan hydrogel to enhance wound healing and observed positive results (59). Johnson determined that carbohydrazide-modified hyaluronic acid hydrogel exhibited high cellular viability and increased cellular growth (60). The ZnO-NP/Chitosan/β-Glycerophosphate composite hydrogels synthesized by Huang et al. exhibited more than 90% cell viability of gingival fibroblasts (61).

#### Release kinetics basic composition gel

The release kinetics of the exosomes incorporated into the hydrogel were monitored using UV-Vis spectroscopy. It provides critical information regarding the release behavior of these exosomes. The absorbance values significantly increased after the addition of the exosomes to the basic composition hydrogel matrix when compared with the hydrogel that contained no exosomes. The absorbance values for both groups (groups 2 and 3) increased over time, which suggests a sustained release of exosomes from the hydrogel, demonstrating first-order kinetics. Group 2 had higher absorbance values when compared to Group 3, which is consistent with a higher concentration of exosomes in Group 2 (62). This signifies the effect of exosome concentration on release kinetics, with a higher concentration leading to higher absorption values. This is most likely due to greater exosome availability. The statistical analysis shows similar results. A statistically significant difference (*p* < 0.05) was found both within and between the groups. A pairwise comparison showed that Group 1 (blank) showed very low absorbance values when compared to groups 2 and 3. Although Group 2 exhibited higher absorbance values when compared to Group 3, the difference was not statistically significant (*p* > 0.05). This shows that both groups followed a similar release pattern. Chen et al. observed a sustained release of exosomes from their synthesized hydrogel, which enhanced bone regeneration in mice (63).

The release kinetics of the exosomes from the hydrogel variants were also monitored via UV-Vis spectroscopy. All the variants showed a similar pattern of release, i.e., the highest rate of release of exosomes was observed at the third minute, after which the rate of release declined. The high initial release rate is possibly due to the high exosome concentration and the breakdown of the hydrogel matrix. After this, there is a stabilization phase, in which there is equilibrium in exosome release. Group CG was an exception to this trend and exhibited a continuous release of exosomes throughout the observation period, which can be attributed to the more stable matrix in the CG group that allowed for a more controlled and delayed release of exosomes (64). However, group CG2 exhibited the highest rate of exosome release at all the observed time intervals, which can be attributed to the more porous structure of CG2 due to the presence of tannic acid in its formulation. This is in accordance with the study by Kaczmarek et al., in which it was observed that hydrogels comprising sodium alginate and tannic acid had a porous structure (65). These differences in exosome release behavior can be beneficial for different hydrogel applications. Group CG can be used for conditions where a sustained release of drugs is required, whereas group CG2 can be used for conditions where a rapid initial release helps in the treatment.

## Conclusion

This study reported the synthesis of a hyaluronic acid/carbomer mucoadhesive hydrogel for oral ulcer drug delivery and its systematic evaluation. Physicochemical and functional tests (using SEM/EDS, ATR-FTIR, rheological, mucoadhesion, and pH-dependent swelling/degradation analyses) were conducted on the basic composition (CG). These tests confirmed a porous, interconnected microstructure suitable for drug loading, characteristic chemical signatures of the constituent polymers, viscoelastic behavior compatible with mucosal application, strong time-dependent mucoadhesion on oral mucosa, and pH-responsive swelling/degradation, which collectively support using the CG as a robust carrier matrix for bioactive payloads. For the release investigation, exosomes were incorporated and their dissolution-mediated kinetics were quantified.

Across the comparative *in vitro* examinations of the variant groups (CG1–CG4), we observed the following: (i) broad antibacterial activity, with the best performance against *E. coli* in CG4 and competitive performance in CG1/CG2; (ii) the strongest activity against *S. aureus* in CG1, followed by CG4; (iii) antifungal activity against *C. albicans* only in CG and CG1, with CG1 exhibiting the larger inhibition zone; (iv) acceptable fibroblast compatibility overall, with CG2 trending the lowest and CG4 the highest at the later time points; and (v) exosome release that scaled with the loading dose in pilot testing and, among the variants, peaked earliest and highest in CG2, whereas CG showed a more gradual, monotonic increase over the short sampling window. After balancing these outcomes for the clinical context of oral ulcers, which often involve a mixed bacterial and fungal bioburden and require a biocompatible, adherent carrier, CG1 (basic gel + EDTA) emerges as the optimal formulation. It provides the most consistent broad-spectrum antimicrobial coverage (including the crucial antifungal effect) while maintaining cytocompatibility, and it supports measurable exosome release for potential regenerative/anti-inflammatory benefit. In contrast, CG2, despite its higher peak release, had weaker biocompatibility and no antifungal effect, and CG4, though demonstrating strong antibacterial activity, lacks antifungal activity. Altogether, CG1 offers the best overall therapeutic profile for an exosome-enabled, mucoadhesive platform for oral ulcers.

In summary, the CG-based hydrogel matrix demonstrated the requisite structure and properties for intraoral use, while CG1 provided the optimal balance of antimicrobial breadth, compatibility, and functional release behavior for translation. The novel hydrogels developed in this study underwent *in vitro* studies, in which only commercial cell lines were used. This meant that this study was ethical, efficient, and scientifically rigorous, reserving indispensable animal testing and clinical trials for later stages.

## Limitations and future recommendations

• Although the *in vitro* results were promising, there is a further need to test the efficacy and safety of the hydrogel in animal studies and clinical trials.
• Due to a limited amount of exosomes available in the study, the sample size for the release kinetics test was small; thus, the power of the test was low. Further testing with a larger sample needs to be conducted.
• Further optimization can be done by adjusting the concentration of the exosomes to further enhance the therapeutic effects or through the addition of an anesthetic to alleviate pain symptoms.
• There is a need to further test the hydrogel in various oral conditions (saliva and pH changes). This will ascertain the hydrogel’s long-term stability and effectiveness over time.
• There is potential for the mucoadhesive gel to be used to treat other mucosal conditions, such as gastrointestinal ulcers.

## Data Availability

The raw data supporting the conclusions of this article will be made available by the authors, without undue reservation.

## Appendix A

Table 2 shows the raw values and means and SDs of the ZOIs of the groups against *C. albicans*.

**Table 2:** Raw replicates and summary statistics.

Group	*n*	R1	R2	R3	Mean	Std. dev.	Std. error	Lower limit of the 95% CI	Upper limit of the 95% CI	Min	Max
CG	3	18.00	21.00	23.00	20.6667	2.51661	1.45297	14.4151	26.9183	18.00	23.00
CG1	3	20.00	25.00	27.00	24.0000	3.60555	2.08167	15.0433	32.9567	20.00	27.00
CG2	3	0.00	0.00	0.00	0.0000	0.00000	0.00000	0.0000	0.0000	0.00	0.00
CG3	3	0.00	0.00	0.00	0.0000	0.00000	0.00000	0.0000	0.0000	0.00	0.00
CG4	3	0.00	0.00	0.00	0.0000	0.00000	0.00000	0.0000	0.0000	0.00	0.00
Total	15				8.9333	11.49824	2.96883	2.5658	15.3008	0.00	27.00
